# Spirituelle Bedürfnisse von Patienten eines Notfallzentrums

**DOI:** 10.1007/s00063-020-00653-8

**Published:** 2020-02-07

**Authors:** E. Frick, A. Büssing, D. Rodrigues Recchia, K. Härtl, A. Beivers, C. Wapler, C. Dodt

**Affiliations:** 1 Forschungsstelle Spiritual Care, Klinik und Poliklinik für Psychosomatische Medizin und Psychotherapie, Klinikum rechts der Isar der Technischen Universität München, Langerstr. 3, 81675 München, Deutschland; 2 Professur Lebensqualität, Spiritualität und Coping, Universität Witten/Herdecke, Witten, Deutschland; 3 Hochschule Fresenius München, München, Deutschland; 4 Klinik für Notfallmedizin, München Klinik Bogenhausen, München, Deutschland

**Keywords:** Intensivmedizin, Krankheitsverarbeitung, Religiöse Bedürfnisse, Existenzielle Bedürfnisse, Spiritual Care, Critical Care, Coping, Religious needs, Existential needs, Spiritual Care

## Abstract

**Hintergrund:**

Spirituelle Bedürfnisse (spB) sind von einer religiösen Gebundenheit prinzipiell unabhängige wichtige Elemente des menschlichen Wesens. Sie können in Belastungssituationen, die z. B. durch das akute Auftreten einer Erkrankung ausgelöst werden, eine Ressource zur Bewältigung der Situation sein. Notaufnahmen sind Bereiche des Krankenhauses mit hoher medizinischer Leistungsdichte, was für Notfallpatienten eine besondere Unsicherheit hervorruft. Die vorliegende Studie untersucht erstmals das Vorliegen spiritueller Bedürfnisse eines Kollektivs von Patienten eines Notfallzentrums.

**Methoden:**

Insgesamt 383 von 479 kontaktierten Patienten füllten die deutschsprachige Version des Spiritual Needs Questionnaire (SpNQ-20) aus und stimmten der Erhebung demographischer und klinischer Daten zu. Die Auswertung umfasste deskriptive Statistiken, Korrelationsanalysen, univariate sowie multiple Varianzanalysen.

**Ergebnisse:**

Bedürfnisse nach innerem Frieden und generative Bedürfnisse (etwas weitergeben, etwas für andere tun) waren stärker ausgeprägt als religiöse (rB) und existenzielle (eB) Bedürfnisse. Es fanden sich keine Zusammenhänge zwischen spB einerseits und Grund der Konsultation, Schweregrad und Anzahl der Komorbiditäten andererseits. Das Alter spielte keine ausschlaggebende Rolle; vielmehr waren die vorhandenen Bedürfnisse, insbesondere die rB, bei Frauen signifikant stärker ausgeprägt als bei Männern.

**Schlussfolgerung:**

Auch in einer Notfallsituation sind Menschen bereit, ihre spirituellen Bedürfnisse zu äußern. Die frühzeitige Erhebung in einer Notaufnahme erfasst auch nichtmedizinische wichtige Aspekte des kranken Menschen und erlaubt die Berücksichtigung der erfassten Bedürfnisse. Ob das auf den weiteren Behandlungsverlauf und das Wohlbefinden der Patienten einen Einfluss hat, müssen weitere Studien klären.

## Abstract (en)

**Background:**

Spiritual needs (spN) are important for human beings—independently of religious affiliation. They can be a resource for coping with stressful situations, e.g., those triggered by the acute onset of a disease. Emergency rooms are hospital departments with high medical performance which may cause a particular insecurity among emergency patients. The present study is the first to examine spiritual needs in a sample of patients in the emergency room.

**Methods:**

A total of 383 out of 479 patients were approached and asked to complete the German version of the Spiritual Needs Questionnaire (SpNQ-20). All consented to the collection of demographics and clinical data. The analysis encompassed descriptive statistics, correlations analysis, univariate and multiple variance analysis.

**Results:**

The needs for inner peace and generative needs (to pass something on to others, to do something for others) were more important than religious (rN) and existential (eN) needs. We did not find a correlation between spN on the one hand and the reason for consultation, the severity, and the number of comorbidities on the other hand. Age did not play a decisive role, rather, patients’ needs, especially rN, were significantly more important among women than among men.

**Conclusion:**

Even in an emergency situation, people are ready to express their spN. Early assessment of these needs exposes important nonmedical aspects of the sick person and helps to consider the assessed needs. Further studies will show whether this has an impact on the further course of treatment and the well-being of the patients.

## Einleitung

Spiritualität ist die Offenheit für die transzendente (über das Messbare, Beeinflussbare hinausgehende) Dimension unseres Lebens. Gerade im Krankheitsfall kann Spiritualität helfen, das Geschehene in das individuelle Leben der Betroffenen einzuordnen [18, 25]. Das American College of Critical Care Medicine unterstreicht die Bedeutung der spirituellen Unterstützung bei kritisch kranken Patienten mit 4 Empfehlungen: 1. Erfassung von spirituellen Bedürfnissen (spB) und deren Einbeziehung in den intensivmedizinischen Behandlungsplan; 2. Spiritual Care-Training für alle Berufsgruppen; 3. Ärzte berücksichtigen die durch Pflege, Seelsorge, Sozialarbeit erfassten spirituellen Bedürfnisse (spB); 4. der Wunsch mancher Patienten, mit ihnen zu beten, wird anerkannt [13, 18]. Die für eine (intensiv-)medizinische Weiterbehandlung erforderlichen Informationen bezüglich der spB von Patienten und ihren Angehörigen sollten frühzeitig, nach Möglichkeit bereits in der Notaufnahme, erhoben werden [18]. Ob und wie und von wem dies umgesetzt werden kann, ist in einer eher säkularen Gesellschaft unklar, insbesondere in der Hightechmedizin mit ihren theoretisch durchgeplanten, aber praktisch doch immer wieder unplanbaren Arbeitsabläufen.

Das Warten auf die Klärung der Bedrohlichkeit einer Gesundheitsstörung und ihrer Behandlungsmöglichkeiten in einem Notfallzentrum stellt in psychosozialer, spiritueller, religiöser und existenzieller Hinsicht eine Krisensituation dar, in der Patienten und Zugehörige professionelle Unterstützung benötigen. Dies ist aber – aus unterschiedlichen Gründen – zumeist nicht gewährleistet. Erschwerend kommt hinzu, dass die Selbst- und die Fremdwahrnehmung von spB auseinanderklaffen können, etwa in der Onkologie [2] oder in der präklinischen Notfallmedizin [22]. Die Sensibilisierung von Ärzten, Pflegenden und anderen Gesundheitsberufen für die spirituelle Dimension findet zunehmende Beachtung, z. B. in der Allgemein- [4] und Palliativmedizin [26], in der Onkologie [3], in Psychiatrie und Psychotherapie [30] und in der Intensivmedizin [18, 23].

Es ist üblich, die Religionszugehörigkeit routinemäßig zu erfassen, und dies ist aktuell in den meisten Krankenhäusern die einzige Routinefrage, die dem Themenbereich wenigstens nahe kommt. Aber eine Religionszugehörigkeit lässt kaum Rückschlüsse auf ihre Bedeutsamkeit im Leben der Person zu [17] und hat nicht notwendigerweise etwas mit der Ausprägung spezifischer spB zu tun. Dennoch kann sie ein möglicher Indikator insbesondere in Bezug auf Probleme und Ressourcen in der Krankheitsverarbeitung (Coping) sein, was für die Arzt-Patienten-Beziehung bedeutsam sein kann. Zum Beispiel kann ein Patient aus religiös-kulturellen Gründen Vorbehalte gegenüber dem Geschlecht einer Ärztin haben, sodass eine vertrauensvolle Zusammenarbeit erschwert ist. Hinter dem soziodemographischen Merkmal „Religionszugehörigkeit“ (das auch verneint werden oder unbeantwortet bleiben kann) verbergen sich sehr verschiedene individuelle und kollektive Facetten nicht nur der Religionszugehörigkeit, sondern der spirituellen, existenziellen, weltanschaulichen Orientierung [17]. Aus praktischen Gründen bedeutsam ist die konkrete Erfassung von existenziellen Bedürfnissen (eB) und spB, die unabhängig von religiöser Orientierung und Weltanschauung beschreibbar und quantifizierbar sind [5, 7]. Abgesehen von der Kostenersparnis durch die Berücksichtigung der spirituellen Dimension [24] verbessert sich die Zusammenarbeit mit dem Patienten, wenn dieser die Wertschätzung seiner spirituellen Orientierung wahrnimmt [21]. Dem Patienten zur Verfügung stehende spirituelle Copingstrategien, z. B. nach einem Verkehrsunfall [1], können wertvolle persönliche Ressourcen darstellen, die möglicherweise professionelle Hilfe vorbereiten.

Zusammenfassend erscheint es wichtig zu sein, SpB bei Patienten zu erfassen. Dies kann entweder im Rahmen der Anamnese unstrukturiert [15] oder strukturiert mithilfe standardisierter Fragebögen geschehen [7]. In beiden Fällen ist auf größtmögliche weltanschauliche Neutralität des Interviewers zu achten, um eine größtmögliche Offenheit bei der Beantwortung der Fragen zu erreichen.

Die vorliegende Studie untersucht erstmals im deutschen Sprachraum spB im Rahmen eines klinischen Notfallzentrums. Dabei geht es um die folgenden Forschungsfragen:

1. Ist eine standardisierte Erfassung von spB unter den besonderen Bedingungen eines Notfallzentrums möglich?
2. Welches Spektrum von spB lässt sich aufzeigen?
3. Sind spB von Notfallpatienten mit Religionszugehörigkeit, Alter, Geschlecht und anderen soziodemographischen Merkmalen assoziiert?
4. Sind spB von Notfallpatienten mit Schweregrad, Diagnosegruppe, Verlaufstyp und anderen Krankheitsvariablen assoziiert?

## Material und Methoden

### Patientenrekrutierung

Befragt wurden Patienten des Notfallzentrums der München Klinik Bogenhausen im Zeitraum vom 3. November bis 09. Dezember 2018. Die Studie war zuvor von der Ethikkommission der Hochschule Fresenius geprüft und genehmigt worden. Nachdem den zu Befragenden der Inhalt und Sinn der Untersuchung erklärt worden war, erklärten sie sich schriftlich zur Teilnahme bereit. Befragt wurden 383 volljährige Patienten und Patientinnen, die nach Einschätzung des ärztlichen und Pflegepersonals aufgrund ihrer klinischen Situation dafür infrage kamen und gute Deutschkenntnisse besaßen. 96 Personen lehnten eine Teilnahme ab (80 % Rücklaufquote).

### Fragebogeninstrumente

Der Fragebogen bestand aus 3 Hauptteilen: einer für soziodemographische Daten, einer für klinisch-dokumentarische Aspekte und einer für die standardisierte Erfassung der spB.

#### Soziodemographische Daten

Von den soziodemographischen Daten wurden Alter, Geschlecht, Familienstatus, Herkunft und Religionszugehörigkeit erfasst. Die Frequenz religiöser Praxis wurde 4‑stufig erhoben (1 – nein, gar nicht; 2 – eher selten; 3 – hin und wieder; 4 – ja, regelmäßig).

#### Klinische Daten

An klinischen Daten wurden die Hauptdiagnosen und das hauptsächlich betroffene Organsystem erfasst, ob ambulant oder stationär behandelt wurde sowie der modifizierte Komorbiditätsindex (CCI; Range, Min, Max, von 0 aufsteigend bis 35; [12]), der Emergency Severity Index (ESI; Range absteigend von 1 [höchste Dringlichkeit] bis 5; [31]) und die Dauer des stationären Aufenthalts. Kommentare und Beobachtungen beim Ausfüllen des Fragebogens wurden dokumentiert.

#### SpB

Die Stärke der spB wurde standardisiert mit dem deutschsprachigen Spiritual Needs Questionnaire (SpNQ-20 mit 20 Items) erfasst. Die 20 Items des Fragebogens werden in 4 Hauptkategorien differenziert [6, 10] und die Stärke des jeweiligen Bedürfnisses in 4 Stufen (0 – nicht, 1 – gering, 2 – mittel, 3 – groß) graduiert:

1. religiöse Bedürfnisse (rB, Cronbachs α = 0,85): selbst beten, mit jemandem beten, dass jemand für einen betet, an einer religiösen Feier teilnehmen, Lesen von spirituellen/religiösen Büchern, sich an eine höhere Präsenz wenden (Gott, Engel usw.);
2. existenzielle Bedürfnisse (eB, Cronbachs α = 0,73): ungelöste Dinge aus dem Leben klären, einen Sinn in der Situation sehen, mit jemandem die Fragen nach dem Sinn des Lebens ansprechen, mit jemandem über die Möglichkeit eines Lebens nach dem Tod sprechen, jemandem aus einem bestimmten Abschnitt des Lebens vergeben, eigene Vergebung;
3. Bedürfnisse nach innerem Frieden (Cronbachs α = 0,75): an Orten der Ruhe und des Friedens verweilen, in die Schönheit der Natur eintauchen, inneren Frieden finden, mit jemandem über Ängste und Sorgen sprechen;
4. Bedürfnisse nach Generativität (Cronbachs α = 0,70): etwas von sich verschenken, jemandem Trost spenden, Lebenserfahrung weitergeben, Gewissheit haben, dass das eigene Leben sinn- und wertvoll ist.

Die interne Konsistenz des SpBQ ist mit Cronbachs α = 0,87 gut (Cronbachs α der 4 Faktoren in diesem Sample: 0,87, 0,73, 0,74 und 0,71).

### Statistische Auswertungen

Deskriptive Statistiken, univariate Varianz- und Korrelationsanalysen wurden mit IBM-SPSS 23 (IBM, Armonk, NY, USA) gerechnet.

Multiple Varianzanalysen (MANOVA) wurden mit dem Software R Studio 3.4.2 berechnet. Dieses statistische Verfahren ist der univariaten Varianzanalyse ähnlich; es unterscheidet sich jedoch hinsichtlich der Anzahl der abhängigen Variablen im Modell, die bei der MANOVA mehr als eine einbezieht und Mittelwertunterschiede mehrerer Einflussgrößen zwischen Gruppen testet.

Ein *p* < 0,05 wird als signifikant erachtet, jedoch wird aufgrund des Einbezugs mehrerer Variablen *p* < 0,01 als bedeutsamer erachtet. In Bezug auf die Klassifizierung der Stärke der beobachteten Korrelationen wird ein r > 0,5 als eine starke Korrelation, ein r zwischen 0,3 und 0,5 als moderate Korrelation, ein r zwischen 0,2 und 0,3 als schwache Korrelation und ein r < 0,2 als keine oder vernachlässigbare Korrelation angesehen.

## Ergebnisse

### Soziodemographische und medizinische Beschreibung

Untersucht wurden 383 Personen. Altersgruppen und Geschlecht sind weitgehend gleichmäßig verteilt (Tab. 1). 77 % der Patienten hatten eine Religionszugehörigkeit (zumeist christlich), 23 % keine. Eine religiöse Praxis hatten 44 % (22 % regelmäßig), jedoch 56 % eher nicht oder gar nicht.

**Table 1:** 

Variablen	%/M ± SD (Range)
*Geschlecht (%)*	
Frauen	52
Männer	48
*Altersmittel, Jahre (M* *±* *SD)*	57,5 ± 21,3 (17–99)
*Altersgruppen, Jahre (%)*	
<41 Jahre	25
41–50 Jahre	13
51–60 Jahre	13
61–70 Jahre	13
71–80 Jahre	21
>80 Jahre	15
*Religionszugehörigkeit (%)*	
Christlich	69
Muslimisch	6
Andere	2
Keine	23
*Religiöse Praxis (%)*	
Nein, gar nicht	43
Eher selten	13
Hin und wieder	22
Ja, regelmäßig	22
*Behandlung (%)*	
Ambulant	48
Stationär	51
*Fachrichtung (%)*	
Innere	29
Chirurgie (inkl. Urologie)	41
Allgemeinmedizin	29
*Organsystem (%)*	
Kardiovaskulär	15
Respiratorisch	6
Nervensystem	15
Gastrointestinal	11
Urogenital	9
Haut	8
Bewegungsapparat	28
Andere	8
*Verlauf (%)*	
Akut	87
Chronisch	7
Exazerbation	3
Rezidiv	3
*Emergency Severity Index (M* *±* *SD)*	3,0 ± 0,7 (2–5)
*Komorbiditätsindex (M* *±* *SD)*	1,1 ± 1,7 (0–13)
*Dauer stationärer Aufenthalt (M* *±* *SD)*	4,1 ± 5,9 (0–57)

*SD* Standardabweichung

Häufigster Vorstellungsgrund waren Schmerzen (67 %), die unterschiedliche Organsysteme betrafen.

Der ESI lag im Mittel bei 3,0 ± 0,7 (Tab. 1); 22 % hatten einen ESI-Score von 2, 58 % einen von 3, 19 % einen von 4 und 1 % einen von 5. Der Komorbiditätsindex (CCI) lag im Mittel bei 1,1 ± 1,7; 55 % hatten einen CCI von 0, 30 % hatten einen von 1–2, 9 % einen von 3 und 6 % einen von 4 und größer. 51 % der Personen wurden stationär weiter behandelt; die Dauer des stationären Aufenthalts betrug im Mittel 4,1 ± 5,9 Tage (Range: 0–57). Im Vergleich zum selben Zeitraum des Vorjahrs fanden sich keine wesentlichen Besonderheiten der jetzigen Stichprobe (nicht dargestellt).

### Ausprägung von spB in Bezug auf Geschlecht, Alter und Religionszugehörigkeit

In der Gesamtgruppe der Patienten waren insbesondere *generative Bedürfnisse* und *Bedürfnisse nach innerem Frieden* ausgeprägt, deutlich geringer *rB* und *eB* (Tab. 2). 33 % hatten keinerlei rB, 23 % keinerlei eB, 4 % keinerlei Bedürfnisse nach innerem Frieden und 2 % keine Generativitätsbedürfnisse. Die in der untersuchten Personengruppe bedeutsamsten Bedürfnisse (mittlere bis große Relevanz) waren: in die Schönheit der Natur eintauchen können (82 %); eigene Lebenserfahrungen weitergeben können (63 %); Gewissheit haben, dass das Leben sinn- und wertvoll ist (59 %); etwas von sich verschenken (56 %); jemandem Trost spenden (55 %); einen Ort der Ruhe und des Friedens finden (54 %).

**Table 2:** 

		Religiöse Bedürfnisse (rB)	Existenzielle Bedürfnisse (eB)	Bedürfnisse nach innerem Frieden	Geben/generative Bedürfnisse	SpNQ20-Score
Alle (*n* = 380)	M	0,73	0,67	1,49	1,59	1,04
	SD	0,84	0,61	0,74	0,73	0,54
*Geschlecht*						
Weiblich (*n* = 197)	M	0,89	0,74	1,57	1,67	1,14
	SD	0,85	0,64	0,76	0,75	0,57
Männlich (*n* = 182)	M	0,57	0,59	1,40	1,51	0,93
	SD	0,79	0,57	0,71	0,69	0,49
F‑Wert		*7,30*	*3,04*	*3,13*	2,96	7,87
*p*-Wert		*0,001*	*0,049*	*0,045*	0,053	<0,0001
*Religionszugehörigkeit*						
Religionszugehörigkeit (*n* = 292)	M	0,88	0,69	1,51	1,63	1,10
	SD	0,86	0,62	0,75	0,71	0,55
Keine Religionszugehörigkeit (*n* = 88)	M	0,25	0,59	1,40	1,45	0,82
	SD	0,50	0,58	0,70	0,77	0,44
F‑Wert		*41,95*	1,73	1,61	*4,45*	*18,68*
*p*-Wert		*<0,0001*	n. s.	n. s.	*0,036*	*<0,0001*

Frauen hatten signifikant stärker ausgeprägte rB, eB und solche nach innerem Frieden als Männer. Für die Altersgruppen ergab sich ein Trend für unterschiedliche Ausprägung von rB (F = 2,21; *p* =0,065) sowie ein signifikanter Unterschied für Bedürfnisse nach innerem Frieden (F = 2,25; *p* =0,049), die beide bei den <40-Jährigen am geringsten waren (nicht dargestellt).

Personen ohne Religionszugehörigkeit hatten signifikant geringere rB, aber auch etwas geringere generative Bedürfnisse (Tab. 2). Die Gruppe der Muslime (*n* = 24) hatte im Vergleich zu den Patienten mit christlichem Hintergrund insbesondere in Bezug auf ihre rB deutlich höhere Ausprägungsscores (1,34 ± 1,10 versus 0,85 ± 0,83).

### Ausprägung von spB in Bezug auf klinische Indikatoren

Die Ausprägung der spB zeigte in Bezug auf den ESI oder die beteiligten Organsysteme keine statistisch signifikanten Unterschiede (Tab. 3). Auch für die Verlaufsform (akut bzw. chronisch) sowie das Merkmal traumatisch vs. nichttraumatisch ergaben sich keine statistisch signifikanten Unterschiede (nicht dargestellt). Weder der ESI, der CCI noch die Dauer des anschließenden stationären Aufenthalts zeigten einen signifikanten korrelativen Zusammenhang mit den spB, wohl jedoch die Ausübungshäufigkeit der religiösen Praxis (Tab. 4).

**Table 3:** 

		Religiöse Bedürfnisse	Existenzielle Bedürfnisse	Bedürfnisse nach innerem Frieden	Geben/generative Bedürfnisse	SpNQ20-Score
All (*n* = 380)	M	0,73	0,67	1,49	1,59	1,04
	SD	0,84	0,61	0,74	0,73	0,54
*Emergency Severity Index (ESI)*						
ESI 2 (*n* = 82)	M	0,68	0,67	1,42	1,56	1,00
	SD	0,82	0,73	0,79	0,72	0,63
ESI 3 (*n* = 217)	M	0,77	0,66	1,54	1,60	1,06
	SD	0,86	0,56	0,69	0,74	0,50
ESI 3 + 4 (*n* = 73)	M	0,71	0,73	1,40	1,60	1,03
	SD	0,77	0,62	0,79	0,72	0,55
F‑Wert		0,39	0,32	1,52	0,08	0,33
*p*-Wert		n. s.	n. s.	n. s.	n. s.	n. s.
*Organsystem*						
Kardiovaskulär (*n* = 58)	M	0,72	0,63	1,50	1,62	1,03
	SD	0,83	0,62	0,75	0,77	0,57
Respiratorisch (*n* = 24)	M	0,93	0,78	1,41	1,51	1,10
	SD	0,88	0,76	0,77	0,56	0,57
Nervensystem (*n* = 54)	M	0,77	0,60	1,41	1,50	0,99
	SD	0,85	0,56	0,73	0,78	0,55
Gastrointestinal (*n* = 41)	M	0,93	0,91	1,76	1,77	1,26
	SD	0,85	0,52	0,71	0,67	0,46
Urogenital (*n* = 34)	M	0,50	0,45	1,51	1,49	0,89
	SD	0,70	0,39	0,69	0,69	0,40
Haut (*n* = 30)	M	0,59	0,75	1,46	1,72	1,04
	SD	0,73	0,69	0,85	0,73	0,55
Bewegungsapparat (*n* = 106)	M	0,72	0,67	1,50	1,59	1,04
	SD	0,85	0,63	0,75	0,70	0,56
Andere (*n* = 30)	M	0,76	0,66	1,22	1,48	0,97
	SD	0,96	0,66	0,57	0,85	0,57
F‑Wert		1,00	1,91	1,55	0,86	1,54
*p*-Wert		n. s.	0,069	n. s.	n. s.	n. s.

**Table 4:** 

	Religiöse Bedürfnisse	Existenzielle Bedürfnisse	Bedürfnisse nach innerem Frieden	Geben/generative Bedürfnisse	SpNQ20-Score
Religiöse Bedürfnisse	1,000	–	–	–	–
Existenzielle Bedürfnisse	0,395^**^	1,000	–	–	–
Bedürfnisse nach innerem Frieden	0,377^**^	0,415^**^	1,000	–	–
Geben/generative Bedürfnisse	0,371^**^	0,394^**^	0,310^**^	1,000	–
SpNQ20-Score	0,777^**^	0,734^**^	0,673^**^	0,675^**^	1,000
Frequenz religiöser Praxis	0,733^**^	0,269^**^	0,326^**^	0,250^**^	0,578^**^
*Klinische Indikatoren*					
Emergency Severity Index	0,045	0,059	−0,006	0,023	0,040
Komorbiditätsindex	0,098	−0,099	0,018	−0,035	−0,002
Dauer stat. Aufenthalt	0,025	−0,007	0,079	−0,076	0,002

**p < 0,01 (Spearman-Rho)

Bestimmte Diagnosen wurden daraufhin gezielt betrachtet. Für Patienten mit Schmerzen (*n* = 249) zeigten sich keine signifikanten Unterschiede gegenüber Patienten ohne Schmerzen (F < 1,0; n. s.). In der Gruppe derjenigen mit Verdacht auf Herzinfarkt (*n* = 25) zeigte sich nur ein Trend für geringere Bedürfnisse nach innerem Frieden (F = 3,60; *p* = 0,059), während sich bei Patienten mit Herzinsuffizienz (*n* = 21) keine signifikanten Unterschiede zeigten (F < 1,95; n. s.). Bei Patienten mit zerebrovaskulären Erkrankungen (*n* = 28) zeigten sich signifikant geringere Ausprägungsscores bei generativen Bedürfnissen (F = 7,25; *p* =0,007) und existenziellen Bedürfnissen (F = 5,45; *p* = 0,020). Bei Patienten mit Lungenerkrankungen (*n* = 24) zeigten sich höhere Scores bei generativen Bedürfnissen (F = 5,66; *p* = 0,018) und ein Trend für höhere eB (F = 3,56; *p* = 0,060).

### Multiple Varianzanalysen

Hinsichtlich der soziodemographischen Daten hatten Alter, Geschlecht und eine fehlende Religionszugehörigkeit einen signifikanten Effekt auf die Ausprägung der spB, während sich für die klinischen Indikatoren keine wesentlichen Zusammenhänge zeigten. Mittels multipler Varianzanalysen (MANOVA) sollte nun die Bedeutung dieser Variablen erörtert werden [29]. Hierbei wurde zunächst auch der ESI berücksichtigt, dann aber aus den Modellen wieder entfernt, da sich kein signifikanter Einfluss zeigte. Relevante Interaktionseffekte zeigten sich nur in wenigen Fällen (z. B. für Alter*keine Religion sowie Geschlecht*Alter*keine Religion bei eB und für Geschlecht*Alter*keine Religion bei generativen Bedürfnissen). Basierend auf diesen Modellen wurde das letzte Modell nur mit den signifikanten Variablen angepasst, wobei die 4 Bedürfniskategorien durch Geschlecht, Alter und fehlende Religionszugehörigkeit erklärt werden sollen. Wie in Tab. 5 dargestellt ist, haben alle 3 Variablen einen signifikanten Effekt in den Modellen: Für die rB ist es insbesondere das Fehlen einer Religionszugehörigkeit; für eB sowie solche nach innerem Frieden und Generativität insbesondere das weibliche Geschlecht.

**Table 5:** 

Variablen	Totale Quadratsumme^a^				*p*-Wert (Pillai^b^)
	Religiöse Bedürfnisse	Existenzielle Bedürfnisse	Bedürfnisse nach innerem Frieden	Geben/generative Bedürfnisse	
Geschlecht	8,88	2,05	2,99	2,36	0,003
Alter	2,99	0,36	1,73	0,14	0,001
Keine Religion	23,41	0,34	0,37	1,59	0,009

^a^Die Werte sind totale Quadratsummen („sum of squares“), die sich auf die statistische Signifikanz der mittleren Unterschiede in Bezug auf die Gesamtvariabilität beziehen. Die Varianz der Gruppen wird in einen einzelnen Wert überführt, wobei die Höhe der totalen Quadratsumme der Größe der Variation in den Mittelwerten entspricht

^b^Der Pillai-Test wurde für die Evaluierung der MANOVA ausgewählt, da er sehr robust ist

## Diskussion

Die in der Einleitung formulierten Forschungsfragen können folgendermaßen beantwortet werden:

1. Die Ergebnisse zeigen, dass Patienten in der Notaufnahmesituation spirituelle Bedürfnisse äußern. Die Befragung der aufgrund ihres Allgemein- und Krankheitszustands interviewbereiten Patienten erwies sich als praktikabel. Die Rücklaufquote von 80 % spricht für eine hohe Akzeptanz.
2. Hinsichtlich des Spektrums der Bedürfnisse zeigte sich, dass *generative Bedürfnisse* und *Bedürfnisse nach innerem Frieden* in der Stichprobe stärker ausgeprägt sind als *rB* und *eB.*
3. Vorhandene spirituelle Bedürfnisse, insbesondere die rB, sind bei Frauen signifikant stärker ausgeprägt als bei Männern. Das Alter der Personen spielt keine ausschlaggebende Rolle.
4. Die erhobenen spB von Personen in der Notaufnahme sind nicht abhängig vom Grund der Konsultation, der notfallmedizinischen Behandlungsdringlichkeit und des akutmedizinischen Ressourcenbedarfs (ESI) oder der Anzahl der Komorbiditäten.

SpB gelten in onkologischen oder palliativen Behandlungssituationen als vergleichsweise plausibel, werden aber darüber hinaus bei chronischen Erkrankungen schon in frühen Krankheitsstadien berichtet [9]. In der Onkologie wird Disstress inzwischen als 6. Vitalzeichen aufgefasst; aufgrund der hohen Bedeutung spirituellen Leidens für die Krankheitsverarbeitung wird grundsätzlich die Erfassung von spirituellem Disstress empfohlen [11]. Allerdings werden als Disstress eher Belastungen erfasst. Hingegen drücken Patienten durch das Äußern von spB mögliche Ressourcen aus. Disstress als Beeinträchtigung der individuellen Lebensqualität muss nicht mit dem objektiven Schweregrad der Erkrankung einhergehen. So fand sich weder bei Patienten mit malignem Melanom [19] noch bei Patienten mit chronischen Schmerzerkrankungen [8] ein Zusammenhang zwischen Disstress und Belastungsgrad. Bei Patienten mit chronischen Schmerz- oder Tumorerkrankungen ließen sich hingegen Zusammenhänge mit Ängstlichkeit, Depressivität und Hoffnungslosigkeit aufzeigen [20, 28], aber auch mit spezifischen Krankheitsinterpretationen [8]. Für die Situation in der Notaufnahme gibt es derzeit kaum vergleichbare Daten. Interessant ist allerdings, dass in der Akutsituation die Krankheitslast, die sich im Komorbiditätsindex ausdrückt und auch in der notfallmedizinischen Behandlungsdringlichkeit, die sich in dem ESI abbildet, nicht mit veränderten spirituellen Bedürfnissen einhergeht. Bei Menschen in Altenheimen zeigte die Ausprägung der entsprechenden Bedürfnisse einen Zusammenhang mit Trauer, emotionaler Müdigkeit und geringer Lebenszufriedenheit [14, 27]. Möglicherweise brauchen kranke Menschen eine ruhige und ausreichend lange Reflexion darüber, welche Ressourcen zur Verfügung stehen, welche Copingstrategien hilfreich sein könnten, welche Bedürfnisse sich daraus ergeben und ob Hoffnung besteht, dass die Berücksichtigung (oder sogar Erfüllung) dieser Bedürfnisse hilfreich für den Coping- und Genesungsprozess sein könnten. Ein für die Notfallsituation charakteristisches reflexives Element dürfte bei den am stärksten ausgeprägten Bedürfnissen darin liegen, dass zum einen die Distanzierung von der Situation gewünscht wird (in die Natur eintauchen und Frieden finden) und zum anderen die Vergewisserung der Sinnhaftigkeit des eigenen Lebens bedeutsam ist (Leben ist wertvoll, Lebenserfahrungen weitergeben, etwas verschenken). Das erste Motiv kann zwar auch als Flucht vor der Situation und den Krankheitssymptomen verstanden werden, ist aber bei den meisten Personen – insbesondere im Disstress – zu finden. Das generative Moment hat etwas mit „Abschiednahme“ in Zeiten der Unsicherheit zu tun, bei dem die Frage nach dem Sinn des eigenen Lebens aufkommt, die Suche nach einem existenziellen Grund, der auch dann noch trägt, wenn die selbstverständlichen Funktionen des eigenen Leibs versagen. Bei dieser Suche könnten seelsorgliche, spirituelle und psychotherapeutische Begleitungen hilfreich sein.

### Limitierungen

Es wird nicht der Anspruch erhoben, dass die vorliegenden Daten repräsentativ für Deutschland sind; die Ausprägung von spB scheint auch von regionalen Besonderheiten mit beeinflusst zu werden, wie ein Vergleich der spB von Bewohnern in Altenheimen aus Schleswig-Holstein [14] und Bayern [27] nahelegt. Das Einschlusskriterium „gute Deutschkenntnisse“ könnte dazu führen, dass Menschen mit Migrationshintergrund und muslimischer Religionszugehörigkeit in der teilnehmenden Stichprobe unterrepräsentiert sind. Einzelne Krankheitsbilder sind zahlenmäßig unterrepräsentiert, sodass die beschriebenen Unterschiede nur hinweisenden Charakter haben; mögliche Hypothesen müssten mit einem größeren Kollektiv gezielt überprüft werden. Sinnvoll wäre es darüber hinaus, die quantitativen Fragebogendaten durch qualitative Interviews zu spirituellen Bedürfnissen zu ergänzen.

### Schlussfolgerungen

Kranke Menschen, die ein Notfallzentrum aufsuchen, bringen vielfältige spB mit, die bisher wenig Beachtung fanden. Ärzte, Pflegekräfte und andere Gesundheitsberufe brauchen – unabhängig von ihrer eigenen Religiosität oder Weltanschauung – eine Basiskompetenz, um diese Bedürfnisse wahrzunehmen und angemessen zu reagieren [16]. Künftige Studien sollten professionelle Kompetenzen und Interventionen evaluieren, um patientenorientiert auf diese Bedürfnisse eingehen zu können. Die Modalitäten einer solchen Unterstützung müssen noch geklärt werden, da Spiritual Care bisher nicht angemessen in Versorgungs- und Abrechnungsmodellen berücksichtigt ist und oft nur als Leistung der konfessionellen Seelsorge verstanden wird.
